# Diagnostic and Prognostic Value of Cerebrospinal Fluid Lactate and Glucose in HIV-Associated Tuberculosis Meningitis

**DOI:** 10.1128/spectrum.01618-22

**Published:** 2022-06-21

**Authors:** Edwin Nuwagira, Kathy Huppler Hullsiek, Samuel Jjunju, Morris Rutakingirwa, John Kasibante, Kiiza Kandole Tadeo, Enock Kagimu, Lillian Tugume, Kenneth Ssebambulidde, Abdu K. Musubire, Ananta Bangdiwala, Conrad Muzoora, David B. Meya, David R. Boulware, Nathan C. Bahr, Fiona V. Creswell

**Affiliations:** a Department of Medicine, Mbarara University of Science and Technology, Mbarara, Uganda; b Department of Biostatistics, University of Minnesota, Minneapolis, Minnesota, USA; c Infectious Diseases Institute, Makerere University, Kampala, Uganda; d Division of Infectious Diseases, Department of Internal Medicine, University of Minnesota, Minneapolis, Minnesota, USA; e School of Medicine, College of Health Sciences, Makerere University, Kampala, Uganda; f Division of Infectious Diseases, Department of Medicine, University of Kansas, Kansas City, Kansas, USA; g MRC-UVRI-London School of Hygiene and Tropical Medicine Uganda Research Unit, Entebbe, Uganda; h Clinical Research Department, London School of Hygiene and Tropical Medicine, London, United Kingdom; University of Mississippi Medical Center

**Keywords:** cerebrospinal fluid, tuberculosis meningitis, chronic meningitis, CSF lactate, HIV

## Abstract

The role of cerebrospinal fluid (CSF) lactate in tuberculosis meningitis (TBM) diagnosis and prognosis is unclear. The aim of this study was to evaluate the performance of CSF lactate alone and in combination with CSF glucose in predicting a diagnosis of TBM and 14-day survival. HIV-positive Ugandan adults were investigated for suspected meningitis. The baseline CSF tests included smear microscopy; Gram stain; cell count; protein; and point-of-care glucose, lactate, and cryptococcal antigen (CrAg) assays. Where CrAg was negative or there was suspicion of TBM, a CSF Xpert MTB/RIF Ultra (Xpert Ultra) test was performed. We recorded baseline demographic and clinical data and 2-week outcomes. Of 667 patients, 25% (*n* = 166) had TBM, and of these, 49 had definite, 47 probable, and 70 possible TBM. CSF lactate was higher in patients with definite TBM (8.0 mmol/L; interquartile ratio [IQR], 6.1 to 9.8 mmol/L) than in those with probable TBM (3.4 [IQR, 2.5 to 7.0] mmol/L), possible TBM (2.6 [IQR 2.1 to 3.8] mmol/L), and non-TBM disease (3.5 [IQR 2.5 to 5.0] mmol/L). A 2-fold increase in CSF lactate was associated with 8-fold increased odds of definite TBM (odds ratio, 8.3; 95% confidence interval [CI], 3.6 to 19.1; *P* < 0.01) and 2-fold increased odds of definite/probable TBM (odds ratio, 2.3; 95% CI, 1.4 to 3.7; *P* < 0.001). At a cut point of >5.5 mmol/L, CSF lactate could be used to diagnose definite TBM with a sensitivity of 87.7%, specificity of 80.7%, and a negative predictive value of 98.8%. CSF lactate was not predictive of 2-week mortality.

**IMPORTANCE** Tuberculosis meningitis (TBM) is the most severe form of tuberculosis, and its fatality is largely due to delays in diagnosis. The role of CSF lactate has not been evaluated in patients with HIV presenting with signs and symptoms of meningitis. In this study, using a point-of-care handheld lactate machine in patients with HIV-associated meningitis, we showed that high baseline CSF lactate (>5.5 mmol) may be used to rapidly identify patients with TBM and shorten the time to initiate treatment with a similar performance to the Xpert Ultra assay for definite TBM. Elevated CSF lactate levels, however, were not associated with increased 2-week mortality in patients with HIV-associated TBM. Due to moderate specificity, other etiologies of meningitis should be investigated.

## INTRODUCTION

Tuberculous meningitis (TBM) accounts for 1% to 5% of tuberculosis cases and remains the most severe form of tuberculosis, with up to 60% mortality in some populations, such as people with HIV in sub-Saharan Africa (1–4). TBM poses a diagnostic challenge due to the paucibacillary nature of the disease, with approximately half of patients treated empirically without microbiological confirmation (5). Making a diagnosis of definite (microbiologically confirmed) TBM requires identification of Mycobacterium tuberculosis in cerebrospinal fluid (CSF) using either smear microscopy for acid-fast bacilli (AFB), mycobacterial culture, or nucleic-acid amplification tests (6). CSF smear microscopy is highly dependent on the microscopist’s expertise and has a sensitivity of less than 20% in most settings (7), while TB culture turnaround times are typically too long to inform clinical practice. The Xpert MTB/RIF Ultra (Xpert Ultra) assay is currently the best, most widely available CSF commercial nucleic-acid amplification test for the diagnosis of TBM, with a run time of 84 min and a sensitivity of 45% to 93% (6, 8, 9). However, in many African settings, a single Xpert instrument often serves a large population, and samples can be queued, prolonging the turnaround time to days (8). Centrifugation of CSF to concentrate bacilli is suggested for optimal results with Xpert, yet such equipment is often not available at many laboratories in low-income settings (10).

Without reliable microbiologic or molecular techniques, CSF white blood cell pleocytosis, high CSF protein (0.5 to 3 g/L), and low CSF glucose (<2.2 mmol/L, or CSF-to-blood glucose ratio of <50%) in a patient with signs and symptoms of meningitis are suggestive of TBM and used in making treatment decisions. However, the same findings could also suggest bacterial or fungal meningitis (7, 11, 12). Abnormally low CSF glucose in particular has long been part of the diagnostic criteria of TBM; however, it may be less accurate in HIV-associated TBM and is not helpful in differentiating TBM from pyogenic meningitis (13–17). In order to obtain better outcomes in TBM, one area that requires improvement is accurate point-of-care diagnosis (13, 18).

CSF lactate is a rapid point-of-care test that has potential as a biomarker to differentiate the etiologies of meningitis in low-resource settings. Recently, CSF lactate has been shown to predict the survival of patients with HIV-associated cryptococcal meningitis in Uganda (19). In a prospective study that enrolled 55 patients with TBM in India, CSF lactate was higher in patients with definite TBM than in those with possible TBM, but it had limited prognostic capacity (20). The diagnostic and prognostic utility of CSF lactate in TBM is currently unclear (21).

We aimed to describe the diagnostic utility of CSF lactate in patients with suspected TBM and explore whether lactate can predict 2-week mortality (22, 23). We evaluated point-of-care CSF lactate and glucose (separately and in combination) in a cohort of predominantly HIV-positive adults with suspected meningitis in Uganda.

## RESULTS

We reviewed data from 667 patients with HIV-associated meningitis in the screening period. The differences in the baseline participant characteristics are described in Table 1. The overall median age was 36 years (interquartile range [IQR], 30 to 43 years), and 59.5% (*n* = 397) were men. There were 166 participants with definite (*n* = 49; 29.5%), probable (*n* = 47; 28.3%), or possible TBM (*n* = 70; 42.2%). One participant was coinfected with TBM and cryptococcal meningitis. Of those without TBM (*n* = 501), 423 had cryptococcal meningitis, and other identified etiologies are shown in Table 1 footnotes.

**TABLE 1:** Baseline clinical characteristics of study participants by TBM case definition^*a*^

Characteristic^*b*^	Value (median [IQR] or *n* [%]) for TBM classification:					*P* value
	Definite	Probable	Possible	None^*c*^	Overall	
No. of participants	49	47	70	501	667	
Age (yrs)	34 (27–37)	37 (30–47)	38 (31–48)	36 (30–42)	36 (30–43)	0.04
Women	20 (40.8)	19 (40.4)	32 (45.7)	199 (39.7)	270 (40.5)	0.82
Wt (kg)	51.0 (50.0–55.0)	54.0 (50.0–60.0)	51.5 (50.0–60.0)	51.0 (48.0–59.5)	51.0 (48.0–60.0)	0.68
GCS < 15	44 (89.8)	39 (83.0)	50 (71.4)	192 (38.9)	325 (49.2)	<0.001
On ART	23 (52.3)	16 (40.0)	28 (46.7)	212 (43.2)	279 (43.9)	0.62
Time on ART (mo)	1.5 (0.4–3.4)	2.0 (0.6–8.9)	11.1 (1.9–32.3)	3.7 (0.8–33.7)	3.4 (0.7–31.7)	0.05
No. of CD4 cells/μL	94 (45–150)	107 (31–263)	150 (56–260)	24 (9–57)	31 (11–84)	<0.001
Headache	46 (93.9)	37 (78.7)	46 (65.7)	444 (88.6)	573 (85.9)	<0.001
Duration of headache (days)	14 (7–21)	21 (12–30)	17 (9–30)	14 (7–30)	14 (7–30)	0.10
Fever present	44 (89.8)	39 (83.0)	55 (78.6)	213 (42.5)	351 (52.6)	<0.001
CSF metrics						
Lactate (mmol/L)	8.0 (6.1–9.8)	3.4 (2.5–7.0)	2.6 (2.1–3.8)	3.5 (2.5–5.0)	3.5 (2.5–5.4)	<0.001
Glucose (mg/dL)	23.4 (18.0–50.5)	71.0 (43.0–100.0)	97.6 (68.0–125.0)	64.0 (40.0–88.3)	64.9 (39.6–93.7)	<0.001
Opening pressure (cmH_2_0)	23 (13–30)	14 (11–22)	13 (9–19)	21 (13–30)	20 (12–29)	<0.001
No. of WBC/μL	48 (4–160)	4 (4–85)	4 (4–4)	4 (4–35)	4 (4–35)	<0.001
CSF WBC < 5 cells/μL	14 (31.8)	27 (60.0)	53 (81.5)	322 (67.1)	416 (65.6)	<0.001
Protein (mg/dL)	105.0 (56.0–184.0)	89.5 (35.0–137.0)	74.0 (32.0–104.0)	58.0 (30.0–104.0)	61.0 (30.0–111.0)	<0.001
Sterile culture	42 (97.7)	41 (100.0)	66 (100.0)	149 (30.8)	298 (47.0)	<0.001
CrAg positive	1 (2.0)	0 (0.0)	0 (0.0)	418 (83.6)	419 (62.9)	<0.001
Days to LP	1 (1–1)	1 (1–1)	1 (1–1)	1 (1–1)	1 (1–1)	<0.001

a Data are median (IQR) or *n* (%). *P* values are Kruskal-Wallis or chi-square.

b ART, antiretroviral therapy; CrAg, cryptococcal antigen; CSF, cerebrospinal fluid; GCS, Glasgow coma score; LP, lumbar puncture; WBC, white blood cell count.

c In those without TBM by the Marais criteria, the diagnoses were cryptococcal meningitis (*n* = 423), cryptococcal antigenemia with no evidence of cryptococcal meningitis (*n* = 26), bacterial meningitis (*n* = 13), and viral meningitis (*n* = 20). Three other cases received empirical TBM treatment but did not meet case definitions, and the remaining cases without TBM did not have a firm alternative diagnosis (*n* = 16).

Most patients presented with fever, headache, and low (<15) Glasgow coma scale (GCS) scores although lower GCS and fever were more common among those with definite, probable, or possible TBM than among those without TBM. The median CSF opening pressure and median CSF white blood cell (WBC) counts were higher in the definite TBM group than in the other groups, although the opening pressure was similar in the non-TBM group, which largely comprised participants with cryptococcal meningitis. The baseline median CSF lactate was higher among persons with definite TBM (8.0 [IQR, 6.9 to 9.8] mmol/L) than in those with probable TBM (3.4 [IQR, 2.5 to 7.0] mmol/L), possible TBM (2.6 [IQR, 2.1 to 3.8] mmol/L), and non-TBM disease (3.5 [IQR, 2.5 to 5.4] mmol/L). Baseline median CSF glucose was also lowest in the definite TBM group (23.4 [IQR, 18.0 to 50.5] mg/dL), followed by the non-TBM (64 [IQR, 40 to 88.3] mg/dL), probable TBM (71 [IQR, 43 to 100] mg/dL), and possible TBM (97.6 [IQR, 68 to 125] mg/dL) groups.

In univariate and multivariate logistic regression analyses (Table 2), the baseline log_2_ CSF lactate was significantly associated with a diagnosis of definite TBM (univariate odds ratio [OR], 9.1; 95% CI, 5.3 to 15.8; *P* < 0.001); even after adjustment for GCS scores of <15, CD4 T cell count (per 25 cells), and CSF WBC counts of ≤5 cells per μL, there remained a significant relationship (adjusted OR [aOR], 8.3; 95% CI, 3.6 to 19.1; *P* < 0.001). Alternatively stated, for every doubling of CSF lactate (mmol/L), there was an 8-fold increase in the probability that the patient had definite TBM. Lower baseline log_2_ CSF glucose was also associated with a diagnosis of definite TBM (odds ratio, 0.346; 95% CI, 0.25 to 0.48; *P* < 0.001), and the relationship became more pronounced after adjustment (aOR, 0.28; 95% CI, 0.16 to 0.48; *P* < 0.001). Those models combining CSF lactate and glucose each predicted definite TBM, though with lesser magnitudes than when used alone (Table 2).

**TABLE 2:** Baseline associations of CSF lactate and CSF glucose with TB meningitis^*a*^^,^^*b*^

Univariate predictor	Data for TBM classification:					
	Definite		Probable		Probable or definite	
	OR (95% CI)	*P* value	OR (95% CI)	*P* value	OR (95% CI)	*P* value
Women	1.015 (0.562–1.835)	0.96	0.998 (0.545–1.825)	>0.99	1.007 (0.648–1.564)	0.97
Age (per 10 yrs)	0.764 (0.564–1.037)	0.08	1.163 (0.883–1.532)	0.28	0.947 (0.766–1.171)	0.62
GCS < 15	10.33 (4.042–26.41)	<0.001	5.574 (2.563–12.12)	<0.001	8.495 (4.625–15.60)	<0.001
CSF WBC > 5 cells/μL	4.582 (2.374–8.844)	<0.001	1.297 (0.697–2.411)	0.41	2.583 (1.639–4.068)	<0.001
CD4 cell count (per 25 cells)	1.071 (1.016–1.130)	0.01	1.166 (1.093–1.245)	<0.001	1.187 (1.117–1.261)	<0.001
CSF lactate	9.144 (5.305–15.76)	<0.001	1.234 (0.844–1.805)	0.28	3.288 (2.407–4.492)	<0.001
CSF glucose	0.346 (0.250–0.478)	<0.001	1.205 (0.895–1.621)	0.22	0.622 (0.498–0.776)	<0.001
Multivariable models						
Model 1						
CSF lactate	8.346 (3.647–19.10)	<0.001	0.644 (0.355–1.169)	0.15	2.311 (1.430–3.734)	<0.001
Model 2						
CSF glucose	0.284 (0.167–0.482)	<0.001	1.322 (0.864–2.022)	0.20	0.577 (0.405–0.823)	<0.01
Model 3						
CSF lactate	6.086 (2.560–14.47)	<0.001	0.707 (0.373–1.340)	0.29	1.920 (1.155–3.192)	0.01
CSF glucose	0.404 (0.237–0.690)	<0.001	1.215 (0.763–1.935)	0.41	0.690 (0.475–1.004)	0.05

a CSF lactate and CSF glucose are on the log_2_ scale; the odds ratio for these predictors is per doubling of the biomarker measurement (mmol/L).

b CSF lactate and CSF glucose are adjusted for GCS < 15, WBC > 5, and CD4 cell count.

We examined the diagnostic performance of categorical cut points for TBM diagnosis. Cut points of >5.5 mmol/L for CSF lactate and <30 mg/dL for CSF glucose were derived based on the values that maximized the area under the receiver operating characteristic (ROC) curve. Using these cut points, CSF lactate showed a sensitivity of 87.8%, a specificity of 80.7%, and a negative predictive value of 98.8% for diagnosing definite TBM, whereas CSF glucose showed a sensitivity of 61.2%, a specificity of 85.9%, and a negative predictive value of 96.5%. When the reference standard of definite/probable TBM was applied, using the same cut point for lactate but <38 mg/dL for glucose, the sensitivity and negative predictive values decreased for both CSF lactate (60.4% and 92.4%, respectively) and glucose (44.8% and 89.5%, respectively) (Table 3).

**TABLE 3:** Associations of baseline CSF lactate and CSF glucose with TB meningitis

CSF parameter	TBM classification	Cut point^*a*^	Sensitivity (%)	Specificity (%)	Negative predictive value (%)
CSF lactate (mmol/L)	Definite	5.50	87.76	80.74	98.81
	Probable	8.00	23.40	90.32	93.96
	Definite or probable	5.50	60.42	81.79	92.48
					
CSF glucose (mg/dL)	Definite	30.00	61.22	85.92	96.55
	Probable	33.00	91.49	19.52	96.80
	Definite or probable	38.00	44.79	79.33	89.53

a Cut points were determined with separate models for lactate and glucose to maximize the area under the ROC curve using the Youden (J) index method.

**FIG 1:**
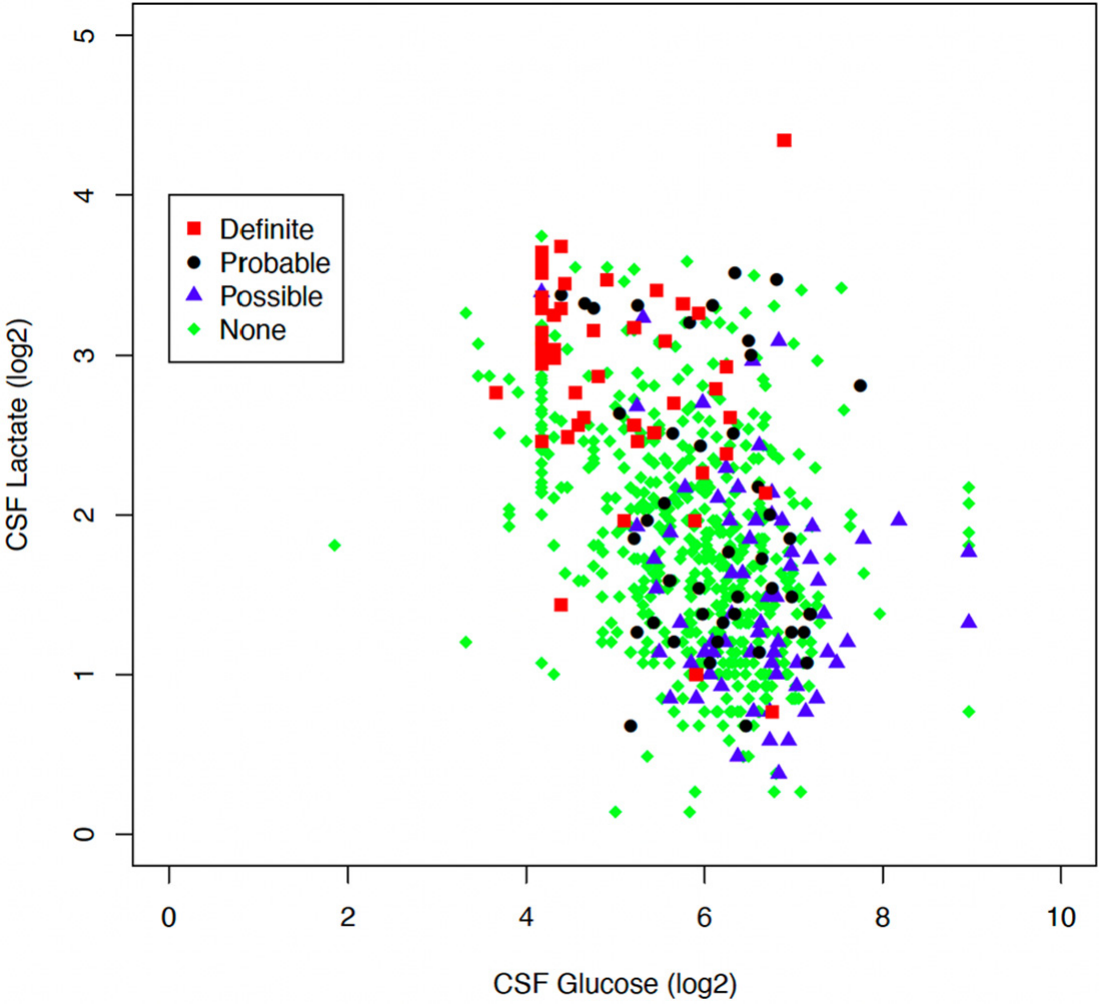
Scatter plot of CSF glucose and lactate by consensus definitions of TBM. The plot shows that the group with definite TBM (red squares) had higher CSF lactate and lower CSF glucose scores than did the groups with probable TBM (black circles) and possible TBM (blue triangles). The green squares show the heterogenous nature of the non-TBM group, with some having high CSF lactate and glucose.

**FIG 2:**
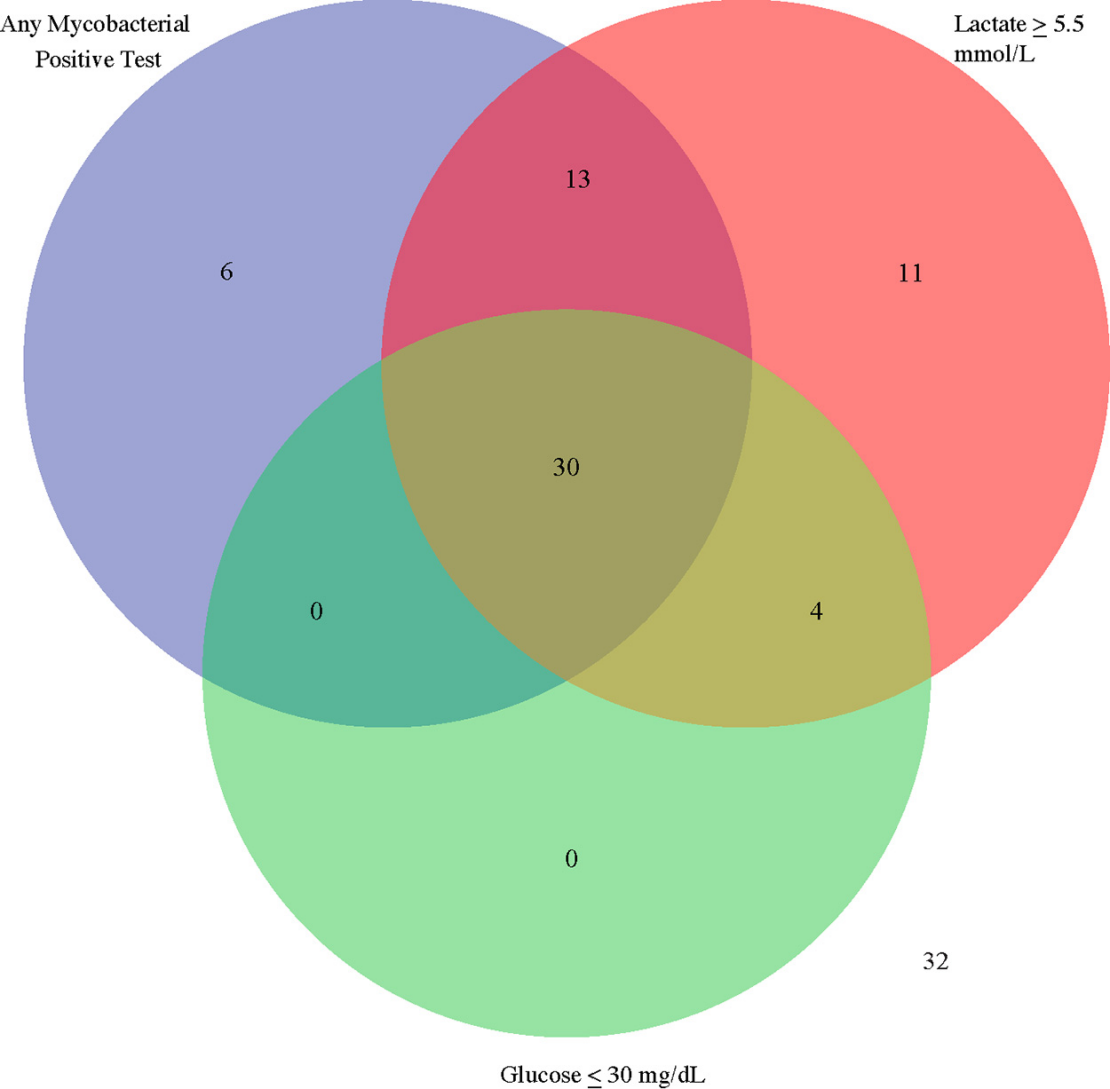
Venn diagram of definite or probable TBM cases (*n* = 96) and diagnostic test results for TBM-specific tests, CSF lactate, and CSF glucose. Participants with any positive mycobacterial test (AFB smear, culture, or Xpert Ultra; *n* = 49) are shown in one circle, those with CSF lactate above 5.5 mmol/L in another, and those with CSF glucose of <30 mg/dL in the last. Fifteen probable TBM cases met the cut points for CSF glucose and/or lactate. Importantly, 32 cases of probable TBM did not have a positive mycobacterial test or meet the cut points for either glucose or lactate.

Figure 1 shows that, in general, the definite TBM group had higher CSF lactate and lower CSF glucose values, while the probable and possible TBM groups tended to have lower CSF lactate and higher CSF glucose values. The “none” or non-TBM disease group had more mixed values, likely owing to the general inadequacy of TBM diagnostics, such that some cases may be included in this group, and the nonspecific nature of CSF lactate and CSF glucose. Figure 2 shows the benefit of adding CSF lactate and CSF glucose measures to the diagnosis of TBM. Among the 96 cases with definite or probable TBM, 49 were definite, and 15 cases met the cut points for CSF glucose and/or lactate; thus, they may have more likely been considered TBM clinically had these cut points been in use. Thirty-two cases of probable TBM did not meet the cut points for CSF glucose or lactate.

The overall 14-day mortality in the definite TBM group was 20.4% (10/49), and in the definite or probable TBM group, it was 20.8% (20/96). CSF lactate did not predict the 14-day mortality of patients with definite TBM (hazard ratio, 1.46; 95% CI, 0.45 to 54.7; *P* = 0.52) or definite/probable TBM (hazard ratio, 1.4; 95% CI, 0.82 to 2.7; *P* = 0.2). Similarly, CSF glucose alone or when modeled in combination with CSF lactate did not reliably predict 14-day mortality (see Text S1 in the supplemental material).

## DISCUSSION

In this study, we examined the diagnostic and prognostic value of baseline CSF lactate among Ugandan adults with suspected TBM. Our study highlights the potential usefulness of CSF lactate in the rapid diagnosis of TBM in people living with HIV. A baseline CSF lactate value above 5.5 mmol/L had a sensitivity of 87.8% in the diagnosis of definite TBM and 60.4% in the diagnosis of definite/probable TBM. This is similar to the sensitivity of the Xpert Ultra assay in some studies (8) and, with immediate results at the bedside, may reduce the time to treatment initiation. However, with a specificity of ~80%, a CSF lactate value of >5.5 mmol/L does not exclude alternative etiologies, including cerebral malaria, cryptococcal meningitis, cerebral injury, subarachnoid hemorrhage, seizures, and ischemia (21, 24–27), hence the need to investigate other etiologies in parallel.

Our results are consistent with two other diagnostic studies. In a Vietnamese cohort of 132 adults, CSF lactate was associated with microbiologic confirmation of TBM (odds ratio, 1.4; 95% CI, 1.1 to 1.7; *P* = 0.001) (28). In a large multisite study in Vietnam and South Africa including 618 adults, of whom 31% were HIV positive, an association between log_2_ CSF lactate and definite TBM remained significant (odds ratio, 3.4; 95% CI, 1.3 to 9.1; *P* = 0.012) after adjustment for baseline CSF findings, HIV status, and geographical location (29).

Although CSF glucose is the most widely used biomarker for TBM currently, we found that a baseline CSF glucose value of <38 mg/dL had only 68% sensitivity and 80% specificity for definite TBM. This is similar to findings from a prospective study done in Peru, a low HIV prevalence setting, where a CSF glucose level of ≤50cmg/dL had a sensitivity of 69.5% and a specificity of 63.5% for diagnosing TBM (16), although in our study, the sensitivity dropped to <45% with definite or probable TBM. Furthermore, adding glucose to lactate did not appreciably improve the performance of CSF lactate alone. Previous studies have shown that low CSF glucose cannot be used to reliably differentiate TBM from pyogenic meningitis (30, 31), and it is possible that some of the patients in the probable TBM group had undiagnosed bacterial meningitis.

The pathogenesis of cerebral ischemia in TBM leading to elevated CSF lactate is ill defined. Some patients with TBM develop vasculitis in the basal meningeal vessels and perforating branches of the middle meningeal artery (24), which may lead to ischemia and brain edema. In such ischemic states, there are shifts from aerobic to anaerobic metabolism in the brain cells, producing lactate (25). TB-infected microglia cells also respond to ischemia by mobilizing glucose and increasing extracellular lactate, which is later used to generate reactive oxygen species and for oxidative phosphorylation (26). This explains the high lactate and low glucose levels in patients with severe forms of TBM. It should, however, be noted that elevated CSF lactate may occur without ischemia as well.

In a recent study of patients with HIV-associated cryptococcal meningitis, high baseline CSF lactate was associated with mortality, yet we were not able to replicate those findings in TBM (19). In a prospective cohort of 21 HIV-negative patients with TBM, high CSF lactate and low CSF glucose were significant predictors of 7-day mortality (27). However, baseline CSF lactate and CSF glucose did not predict 14-day (or 7-day) survival in our study. Other factors that affect cerebral oxygen delivery, such as baseline blood pressure and anemia, which would contribute to high CSF lactate and low glucose in patients with chronic meningitis (32) and lead to poor outcomes, were not measured.

Our findings are most generalizable to HIV-associated TBM. The external generalizability of our findings remains to be examined in other settings, particularly those with lower HIV prevalence among the study population. Further studies are also needed to explain what, if any, relationships exist between CSF lactate and other host markers of TBM disease (such as tumor necrosis factor alpha [TNF-α], interleukin-6, and tryptophan, among others) in people living with HIV (33).

Our study has several limitations. First, CSF glucose is one criteria used in assigning the TBM case definitions; therefore, collinearity is present in the glucose analyses that utilize these definitions. Yet glucose is but one of many factors in the TBM cases definitions, and so, while there is likely some effect, it is unlikely that this limitation significantly changes the overall findings as they pertain to CSF glucose and TBM in this analysis. The retrospective nature of the analysis is another limitation in that it does not allow us to ascertain whether real-time clinical benefit occurs by utilizing the point-of-care tests as they are conducted. A further limitation is the nonspecific possible TBM group, which is heterogenous in an HIV-positive population and likely overstates the probability of TBM being a realistic possibility in many of these cases.

### Conclusion.

Our data showed that point-of-care CSF lactate is a useful biomarker to aid diagnosis of TBM at the bedside that may help reduce the time to treatment initiation while other test results are awaited. A cut point of >5.5 mmol/L has a sensitivity similar to the CSF Xpert Ultra assay, although the low specificity means that investigation for other potential causes of presentation should be pursued in the absence of confirmation of M. tuberculosis infection. Importantly, even combining CSF lactate with the Xpert Ultra assay would not be adequate to rule out TBM in our patient population. More research is needed to better understand the role of lactate at the host-pathogen interface during Mycobacterium tuberculosis infection. Inclusion of CSF lactate when developing clinical diagnostic scoring systems for the TBM consensus case definition should be considered.

## MATERIALS AND METHODS

### Study setting and study population.

We retrospectively reviewed data from patients who presented with symptoms of a central nervous system (CNS) infection to Kiruddu National Referral Hospital and Mbarara Regional Referral Hospital, Uganda, between April 2017 and March 2022. Patients underwent lumbar puncture as part of the screening process for three clinical trials (34–36) and an observational cohort focused on meningitis diagnostics (9). Following informed consent, a diagnostic lumbar puncture was performed with CSF collection. Participants were followed until hospital discharge, 2 weeks in hospital, or death, whichever came first. For participants who were discharged prior to completion of 2 weeks, the study team ascertained their status through a phone call. TBM diagnosis was based on the published 2010 uniform case definition (37), where definite TBM was defined as microbiological confirmation of Mycobacterium tuberculosis in CSF using microscopy, mycobacteria culture, or nucleic-acid amplification tests, probable TBM was defined as a total diagnostic score of ≥12 points in the presence of brain imaging and ≥10 points in the absence of brain imaging, and possible TBM was defined as a diagnostic score of 6 to 9 points without brain imaging or 6 to 11 points with brain imaging. Patients were classified as non-TBM if another diagnosis was confirmed, such as cryptococcal meningitis, or if their diagnostic score was <6 points.

### CSF analysis.

Upon lumbar puncture, the following point-of-care CSF testing was performed: (i) glucose using a handheld automated glucometer (LifeScan, Malvern, PA, USA), (ii) cryptococcal antigen (CrAg) via lateral flow assay (LFA) (IMMY, Norman, OK, USA), and (iii) lactate via a handheld lactate reader (Nova Biomedical, Waltham, MA, USA). We also sent CSF for routine analysis that included total protein, total white cell count and differentials, Gram and Ziehl-Neelsen stains, Xpert Ultra, and routine bacterial culture. Where the above-described tests were nondiagnostic, we performed a FilmArray meningoencephalitis multiplex PCR (Biofire, Salt Lake City, UT), which tests for 14 common bacterial, fungal, and viral CNS pathogens, not including Mycobacterium tuberculosis.

### Statistical analysis.

Our primary objectives were to evaluate the diagnostic accuracy of CSF lactate and glucose for TBM, as well as their relationship with 2-week mortality. To describe the baseline demographic and clinical characteristics, we compared medians across the definite, probable, possible, and non-TBM groups, using Kruskal-Wallis or chi-square tests as appropriate. Logistic regression models with continuous CSF lactate and CSF glucose on the log_2_ scale (separately and combined) were used to predict the TBM status for two different comparisons: (i) participants with definite TBM compared to those with probable, possible, or non-TBM disease; and (ii) participants with definite/probable TBM compared to those with possible or non-TBM disease. The adjusted models included covariates for Glasgow coma scale (GCS) scores of <15, CD4 count (per 25 cells), and CSF white blood cell counts of ≤5 cells/μL. Receiver operating characteristic (ROC) analysis was performed to determine the optimal cut point values for baseline CSF lactate and CSF glucose to maximize the area under the curve for the two events and evaluate the sensitivity, specificity, and negative predictive values for the cut point presented. All statistical analyses were performed using SAS version 9.4 (SAS Institute, Cary, NC, USA).

### Ethical approval.

All participants or their legal representatives (where the patient had altered mentation) provided written informed consent. All studies were approved by the Mulago National Research Ethics Committee, the Uganda National Council of Science and Technology, and the University of Minnesota.
