# Supplementary material for: Diagnostic and Prognostic Value of Cerebrospinal Fluid Lactate and Glucose in HIV-Associated Tuberculosis Meningitis
Source: Microbiol Spectr. 2022 Jun 21;10(4):e01618-22. doi: 10.1128/spectrum.01618-22 (PMC9430741; doi:10.1128/spectrum.01618-22)
Supplement: Supplemental file 1 — Supplemental material. Download spectrum.01618-22-s0001.pdf, PDF file, 0.02 MB [file spectrum.01618-22-s0001.pdf]

Appendix Table 1: Mortality Predicted by Baseline CSF Lactate and Glucose

	TBM Cohort			
	Definite		Definite or Probable	
Number of People	49		96	
Number of deaths within 7 days	6		16	
Number of deaths within 14 days	10		20	
	HR (95% CI) Among Those With Definite TBM	P-value	HR (95% CI) Among Those With Definite or Probable TBM	P-value
7-day Mortality				
CSF Lactate	0.87 (0.22, 3.41)	0.84	1.24 (0.65, 2.36)	0.51
CSF Glucose	0.61 (0.18, 2.03)	0.42	1.24 (0.73, 2.10)	0.42
14-day Mortality				
CSF Lactate	1.46 (0.45, 4.74)	0.52	1.42 (0.82, 2.47)	0.21
CSF Glucose	0.63 (0.27, 1.48)	0.29	1.01 (0.65, 1.56)	0.98

Univariate models with CSF Lactate and CSF Glucose on the Log_2 scale. Hazard ratios are per doubling of the biomarker measurement.
